# The Importance of Mapping Determinants, Attitudes and Beliefs of Vaccine Hesitancy in the Great Challenge of Compulsory Childhood Vaccination: Comment on "Convergence on Coercion: Functional and Political Pressures as Drivers of Global Childhood Vaccine Mandates"

**DOI:** 10.34172/ijhpm.2023.7614

**Published:** 2023-06-27

**Authors:** Federica Guaraldi, Marco Montalti, Davide Gori

**Affiliations:** ^1^IRCCS Istituto delle Scienze Neurologiche di Bologna, Bologna, Italy; ^2^Unit of Hygiene, Department of Biomedical and Neuromotor Sciences, Public Health and Medical Statistics, University of Bologna, Bologna, Italy

**Keywords:** Vaccine Hesitancy, Compulsory Vaccination, Childhood Vaccinations

## Abstract

Vaccine hesitancy (VH) has risen significantly during the COVID-19 pandemic, becoming a major global health concern. VH is characterized by the delay or refusal of vaccination despite its availability. Various frameworks have been developed to understand the complex factors influencing VH, with attitudes, beliefs, and external influences being the most significant. The surge in VH has reignited the debate on the best approach to address it: persuasive/ educational or coercive. Attwell and Hannah studied the political and social reasons behind the adoption of mandatory vaccination in four jurisdictions (Italy, France, Australia, and California) due to declining vaccine coverage below the safety threshold. However, these methods may foster parental disbeliefs and opposition to vaccination campaigns. To combat VH, it is crucial to systematically assess its determinants within specific contexts and population groups. Increasing awareness about vaccination benefits, engaging with social media, and employing tailored strategies can foster spontaneous adherence to vaccination programs, eliminating the need for coercive measures.

 Vaccines and vaccination programs have proven to be one of the most effective tools for eradicating, mitigating and controlling life-threatening infectious diseases. It is estimated that 2 to 3 million deaths are prevented by vaccination campaigns every year,^1^ not considering the recent significant reduction of death and morbidity associated with COVID-19.^2^ They represent the most cost-effective public health policies in children, and, whenever evidence-based public health strategies are applied, even the most hard-to-reach and vulnerable populations^3^ as well as subgroups with peculiar needs (ie, pregnant women, travelers, immunocompromised patients, etc) can take advantage.^4,5^ To maximize vaccine effectiveness in reducing infections spreading, the coverage in the pediatric population must remain above a specific threshold, determined by the type of vaccine and pathogen.

 Somehow paradoxically — considering the accumulating evidence on the safety and efficacy of various vaccines, created using different technologies, and administered to large cohort of adults, children, elderly, pregnant women, and ‘frail’ patients —, the number of people reporting concerns and misperception on vaccination is increasing. In particular, parents/guardians are suspicious about childhood vaccinations, both for vaccines in use for several years/decades, and the recently introduced COVID-19 vaccines. The introduction of compulsory vaccination is a long lasting debate which has occurred in the public debate even before COVID-19 crisis. During the pandemic period a sort of convergence towards coercion has been observed in many western countries, as debated in the recent paper by Attwell and Hannah.^6^

 Parental attitude towards mandatory vaccination in the pre-COVID-19 era in the different countries has been systematically analyzed by Gualano et al.^7^ The rate of parental support to mandatory vaccination varied from 53% to 97%, and was influenced by the type of vaccination program, country income, religious or personal beliefs. Overall, 90% of the parents supported children vaccination independently from the vaccination policy. On the other hand, mandatory vaccination seemed to increase opposition by some parents that believed that vaccination should be a matter of parental choice only.

*Vaccine hesitancy (VH)*, defined by the SAGE working group as “delay in acceptance or refusal of vaccination despite availability of vaccination services”^8^ has become a serious threat to public health strategies aimed at warranting an adequate vaccination coverage worldwide, deserving inclusion among major health concerns by the World Health Organization (WHO) in 2019.^9^

 Tackling VH is extremely complex because of the multitude and heterogeneity of underlying individual and context geographical, cultural, educational, religious, socio-economic, and behavioral determinants, making it an ever-changing phenomenon.^8-10^ For example, VH and outright refusal in high income countries, with well-resourced vaccination programs, could depend on inadequate/poor immunization program communication, while in low- and middle-income countries, from the scarce communication resources limit the ability of vaccination campaign to reach the community and counteract disbeliefs on vaccines.^8^ Also, as it will discussed afterwards, other factors such as pressure of mass media are among the important factors to drive VH.^11^

 Moreover, vaccination, like other medical acts, requires the release of informed consent by the patient for him/herself or his/her child, based on his/her *intention*. Intention is mediated by the ‘subjective norm’ that each person creates for a specific *behavior* and is mediated by the attitudes towards and beliefs about it.^12^ *Attitudes* and *beliefs* towards vaccination are influenced by several context specific factors, skillfully described by the 5C — Confidence, Complacency, Convenience, Collective Response, and Calculation — model. Different taxonomies and archetypes of people who refuse vaccinations have been proposed,^13^ that for research purposes could be grouped into two main categories: those for whom attitudes and perceptions like fear of adverse events following immunization, needle phobia, and perception of a low risk of contracting the disease or experiencing serious events- influence beliefs towards vaccinations; and those for whom religious or political beliefs, and/or prejudices influence the attitudes and perceptions. This classification is not merely speculative but implies the adoption of different strategies to improve vaccine confidence and adherence. Specifically, the implementation of educational programs, including information campaign on the real risk associated with vaccination, and the introduction of compulsory vaccinations (in this way, vaccination would no longer be an individual choice, but would be determined by the government, to which all responsibility will be delegated, reliving the parent/guardian from personal fears) appears effective with the first category of hesitant people, but worthless and even counter-productive for the increase of the contentiousness or rebellious spirit of people included in the second category, for whom well-designed psycho-attitudinal approaches are, instead, advised.

 COVID-19 pandemic has boosted the longstanding debate on strategies to be adopted to increase vaccine confidence and adherence, making it a public health and government priority. Under-vaccination is an extremely complex problem — encompassing delay and refusal driven by social, cultural and other contextual factors, together with failure to reach some populations —, and various educational and coercive measures had been adopted by the various countries, varying according to the public health policies, the type of disease to be tackled, and other social and contextual factors.^10^

 At this regard, Attwell and Hannah^6^ highlights the convergence since 2015 of California, Australia, France, and Italy on coercive measures to increase vaccine adherence in the pediatric population, by expanding/heightening consequences (ie, refusal of educational enrolment, fines, and loss of financial entitlements) and removing exemptions for parents/guardians of unvaccinated children. These measures appear in contrast with persuasive measures adopted by the governments of other industrialized countries, not supported/opposed by leading international non-governmental organizations, global vaccination networks and communities of practice. Moreover, in the authors’ opinion, strategies were heterogeneous and uncoordinated among countries, not converging in the same direction of change during a defined period, possibly because of diverse local factors, suggesting the role of local political pressure on central government decisions and, in general, the interplay between functional and political aspects in vaccine mandates.

 In Italy, compulsory vaccination for ten infectious diseases (ie, diphtheria, tetanus, pertussis, poliomyelitis, Haemophilus influenzae type B, hepatitis B, measles, mumps, rubella, and chicken pox) in children was introduced in July 2017, to restore the appropriate threshold coverage rates defined by the National Vaccine Plan (Piano Nazionale Prevenzione Vaccinale) after measles outbreak, and resolve the confusion generated by the division between ‘compulsory’ and ‘recommended’ vaccinations in previous legislation. This strategy proved to be effective as it increased vaccine coverage for the hexavalent vaccine against diphtheria, tetanus, pertussis, poliomyelitis, Haemophilus influenzae type B and hepatitis B of 1%, and for measles, mumps, and rubella vaccine of 2.9%, from June to October 2017, and allowed to reach the recommend immunization threshold in 2018.

 Similarly, in France the tremendous increase of VH (caused, at least in part, by the confusion made by the combination of ‘voluntary’ and ‘mandatory’ vaccinations) together with a measles epidemic in 2017 led the government to impose 11 vaccinations in children to access care, early education and school.

 In Australia a comprehensive mandate covered all recommended vaccines. The shift toward a more restrictive pattern was not guided by infectious outbreak or vaccine coverage reduction, but by people spontaneous mobilization, guided by vaccine refusal’s potential threat in children drove the change in vaccine policy, pressuring the government to remove Conscientious Objection, that allowed medical exemption from vaccination. Media played a pivotal role first by speaking about potential health danger in regions with lower vaccine coverage, then presenting vaccine refusers as selfish and dangerous, strongly stimulating government intervention. In 2015 Conscientious Objections were abolished, shifting Australia to a restrictive mandate, after which families not vaccinating children for non-medical reasons lost cash payments and children subsidies.

 In California, a measles outbreak sourced to Disneyland at the end of 2014 was determinant in bringing community and political attention on the need of a more coercive mandate for vaccination. The country laws already required children to be vaccinated against a range of diseases to enroll in school but permitted non-medical exemptions. Through skillful mobilization and framing, as well as sustained lobbying and advocacy, California’s exemption abolitionists convinced the political class to hold the line in the face of unprecedented opposition from vaccine refusing families, and in July 2015 an ad hoc law was signed.

 The four case studies reported by Attwell and Hannah points out two main aspects related to vaccine policies. First, coercive measures can be elicited by people call-to-action (bottom-up model) or directly imposed by governments (top-down model). Independently by the driving factors, these measures are adopted whenever an epidemiological threat secondary to the reduction of vaccine coverage for a defined infectious disease is foreseen by health authorities. Nevertheless, as per other public health practices, it would be desirable to reach in the field of vaccine strategies, the so-called ‘ethical equilibrium’ among parental autonomy, state laws, and the necessary levels of vaccine coverage, thus avoiding the need for coercive acts.^13^ Second, although necessary and decisive to restore appropriate vaccine coverage for a defined disease to safeguard population health, these measures risk to foster the opposition by people refusing vaccines for religious or political beliefs, and/or prejudices, that represent an important fraction (although not all) of the hesitant population, generically called ‘no-vax,’ and that mostly exploit social media, often with purposely created communication channels, to easily disseminate anti-vaccine conspiracy beliefs and related contents.^14^

 We recently observed that parents/guardians who gather information through official sources are significantly more prone to vaccine their children, while those that mostly refer to social media tended to be hesitant.^15^

 Therefore, new strategies aimed at enhancing the awareness on the existence and correct consultation of official data sources, as well as at disseminating evidence-based information on the health risks associated with infectious diseases and potential benefits of vaccine administration using targeted strategies on different social media, are strongly advocated to increase population health literacy and, therefore, spontaneous informed adherence to vaccination. The systematic monitoring and reporting of adverse events potentially related to vaccination on national ground would further help to tackle the growing skepticism and disbelief of parents/guardians related to their children vaccination.^10^

 Evaluating attitudes towards mandatory vaccination, in particular in parents/guardians, is the keystone to better understand vaccination-related issues and plan suitable strategies to improve immunization coverage in the pediatric population. For the great heterogeneity of the determinants of VH, studies performed in specific context and examining sub-populations groups, to be repeated over time appear fundamental, and could be realized by the implementation of dedicated local and national observatories. Data collection and analyses will consequently guide new public health strategies, to counteract VH and, therefore, avoid the need of urgent measures, like coercive vaccination. Finally, it must be pointed out that VH is a complex issue, and no standalone strategy can address it completely. Despite the complexity of VH and the broad range of its determinants which still need to be targeted and counteracted, social media engagement activities, public health programs targeted towards the increase of population awareness about benefits of vaccination, and carefully tailored strategies addressing VH determinants will be able to bring out the desired change along with complex political decisions such as the coercive measures debated in the paper.

## Ethical issues

 Not applicable.

## Competing interests

 Authors declare that they have no competing interests.
